# A Positive Control for Detection of Functional CD4 T Cells in PBMC: The CPI Pool

**DOI:** 10.3390/cells6040047

**Published:** 2017-12-07

**Authors:** Annemarie Schiller, Ting Zhang, Ruliang Li, Andrea Duechting, Srividya Sundararaman, Anna Przybyla, Stefanie Kuerten, Paul V. Lehmann

**Affiliations:** 1 Research&Development Department, Cellular Technology Limited, Shaker Heights, OH 44122, USA; annemarie.schiller@student.uni-halle.de (A.S.); ting.zhang@immunospot.com (T.Z.); ruliang.li@immunospot.com (R.L.); duechting.andrea@googlemail.com (A.D.); svidya.sraman@gmail.com (S.S.); anna.przybyla@immunospot.com (A.P.); 2 Department of Cancer Immunology, Chair of Medical Biotechnology, Poznan University of Medical Sciences, 61-701 Poznan, Poland; 3 Institute of Anatomy and Cell Biology, Friedrich-Alexander University Erlangen-Nürnberg, 1054 Erlangen, Germany; stefanie.kuerten@fau.de

**Keywords:** PBMC quality assessment, PBMC cryopreservation, CD4 cell function, ELISPOT, ImmunoSpot, immune monitoring

## Abstract

Testing of peripheral blood mononuclear cells (PBMC) for immune monitoring purposes requires verification of their functionality. This is of particular concern when the PBMC have been shipped or stored for prolonged periods of time. While the CEF (Cytomegalo-, Epstein-Barr and Flu-virus) peptide pool has become the gold standard for testing CD8 cell functionality, a positive control for CD4 cells is so far lacking. The latter ideally consists of proteins so as to control for the functionality of the antigen processing and presentation compartments, as well. Aiming to generate a positive control for CD4 cells, we first selected 12 protein antigens from infectious/environmental organisms that are ubiquitous: Varicella, Influenza, Parainfluenza, Mumps, Cytomegalovirus, *Streptococcus*, *Mycoplasma*, *Lactobacillus*, *Neisseria*, *Candida*, Rubella, and Measles. Of these antigens, three were found to elicited interferon (IFN)-γ-producing CD4 cells in the majority of human test subjects: inactivated cytomegalo-, parainfluenza-, and influenza virions (CPI). While individually none of these three antigens triggered a recall response in all donors, the pool of the three (the ‘CPI pool’), did. One hundred percent of 245 human donors tested were found to be CPI positive, including Caucasians, Asians, and African-Americans. Therefore, the CPI pool appears to be suitable to serve as universal positive control for verifying the functionality of CD4 and of antigen presenting cells.

## 1. Introduction

For decades, immune monitoring has been largely confined to assessing humoral immunity via the detection of serum antibodies. As antibody molecules are rather stable in serum, sample shipment from clinical sites to central test facilities, storage, and batch testing has been a readily manageable task. Measurements of cellular immunity, in contrast, requires functional tests done with viable peripheral blood mononuclear cells (PBMC) that are highly sensitive to optimized ways of isolation, shipment, and storage, as well as cryopreservation and thawing, whereby all these measures can critically impair the functionality of the cells [1,2,3]. Successful T cell immune monitoring is, therefore, critically dependent on the integrity of the PBMC test samples. Only recently have protocols and test methods advanced to a point where also cellular immune monitoring can deliver robust results [4,5], and can be validated [6] for large scale clinical trials. Identifying positive control antigens that permit the verification of the functional integrity of each PBMC test sample are a critical part of this process.

A positive control for testing CD8 cell integrity in PBMC has been introduced, and is widely used [1]. It consists of a pool of short peptides that correspond to immune-dominant determinants recognized by CD8 cells of three viruses that commonly infect humans by adulthood, namely, cytomegalo-, Epstein-Barr-, and flu virus.

CEF peptides do not test for the functionality of CD4 cells and of the antigen presenting cells (APC) that are required for processing and presenting antigen to CD4 cells. When added in solution to the test medium, short peptides like those contained in the CEF pool will bind directly to HLA-class I molecules on the surface of all Class I positive cells in PBMC (that is, all cell types contained in PBMC including CD8 cells themselves) and these peptides will be recognized by the peptide-specific CD8 cells without the need for additional antigen processing [7,8]. This is because antigen-recognition by CD8 cells has evolved for the detection of antigens actively biosynthesized in cells, primarily for the identification and killing of cells that replicate viruses. As (viral) proteins are synthesized in cells, their peptide fragments participate in the endoplasmic reticulum in the assembly of HLA-class I molecules and the peptide-loaded Class I molecules are transported for display on the (infected) cell’s surface [9]. The addition of antigenic peptide determinants in solution to PBMC bypasses this physiological pathway for antigen presentation to CD8 cells: the added peptide displaces third party peptides on the cells’ surface that had been loaded intra-cellularly, and is now displayed to CD8 cells for recognition [9].

The use of peptides as antigens for CD8 immune monitoring, including CEF peptides entails the challenge to accommodate the HLA-class I allele diversity of the human test population. Therefore, either the peptides need to be customized to the major immunohistocompatibility (MHC) Class I alleles expressed in the different test subjects, or sizable random peptide pools need to be used in order to cover the relevant antigenic peptides by chance. Establishing the CEF peptide pool has relied on the former approach, accommodating the HLA-class I alleles that are common in Caucasians, but leaving underrepresented rarer alleles in Caucasians, and prevalent alleles in other races. Subsequently, as our data reported here will confirm, the CEF peptide pool can relative frequently fail as a positive control for CD8 cells.

While immune monitoring of CD8 cells, for the above-mentioned reasons, requires the use of peptide antigens with all of the challenges of the peptide approach, in vitro testing of antigen-specific CD4 cells can, and ideally does, utilize the entire antigen. CD4 cells require the antigen to be presented on HLA-class II -positive APC, that in PBMC are primarily dendritic cells, macrophages, and B cells. Such APC internalize protein antigens from their extracellular environment, i.e., the cell culture medium, via pinocytosis or phagocytosis, and transfer them into their lysosomes. While the antigen undergoes proteolytic digestion there through lysosomal proteases, peptide fragments of the antigen are generated and transferred to a specialized vesicular compartment where these peptides are loaded into HLA-class II molecules. The APC then transfers the HLA-class II/peptide complexes to its cell surface, displaying it for recognition by CD4 cells [9]. A major advantage of being able to use protein antigens for CD4 cell diagnostic vs. utilizing peptides is that all relevant peptide fragments/determinants of the antigen are generated through the physiological antigen processing and presentation process. Each of the three HLA-class II loci in humans underlies considerable polymorphism, resulting in unique HLA-class II types. Subsequently, dictated by each subjects’ HLA-type, the selection and presentation of antigenic determinants is highly individualized. For CD4 cells, the fine tailoring of the relevant peptides to each test subjects’ Class II alleles occurs naturally after the addition of the entire protein antigen. Importantly, the use of protein antigens, in addition to CD4 cells, also tests the functionality of the APC. On the down side, large protein complexes are more likely to activate pattern recognition receptors than short peptides. Thus, for each protein antigen used for CD4 cell stimulation it needs to be established whether it only activates CD4 cells, or also stimulates cells of the innate immune system.

In this study, we have set out to establish a positive control for CD4 cells, that is, to identify antigens that would stimulate a CD4 cell recall response in most human test subjects. Instead of using peptides, we aimed to identify whole antigens that in theory should work across all HLA-types, for all individuals of all human races. As a requirement for antigen processing and presentation, in addition to CD4 cells, such a positive control would also establish the functionality of antigen processing and presentation in HLA-class II positive APC.

## 2. Materials and Methods

### 2.1. PBMC Donors

Cryopreserved PBMC from 245 healthy donors (age 22–45) were selected from the ePBMC library (CTL, Shaker Heights, OH, USA: www.immunospot.com/ImmunoSpot-ePBMC). The race distribution of these donors was 26 African American, 9 Asian, 79 Caucasian, 129 Hispanic, and two American Indians. These donors were recruited by Hemacare (Van Nyus, CA, USA) and their PBMC isolated by leukapheresis using Hemacare IRBs. The PBMC were then cryopreserved at CTL and stored in vapor liquid nitrogen until testing [10]. Thawing, washing, and counting of the cryopreserved cells was done according to an optimized protocol [3]. After thawing, viability of PBMC was invariably greater than 90%. Within maximally 4 h after thawing, the cells were transferred into the ELISPOT test system. Thus, the thawed cells were not “rested”, as we have established that resting does not improve the CD4 or CD8 cell performance of cryopreserved PBMC when handled according to the above protocols [11].

In select experiments, CD4+ or CD8+ T cell subsets were depleted from PBMC using a magnetic bead selection kit (Stem Cell Technologies, Vancouver, BC, Canada). The depletion assay was performed according to manufacturer’s instructions.

### 2.2. Antigens

For screening, 13 viral and bacterial antigens were tested in an IFN-γ ImmunoSpot^®^ assay. All viral antigens were purchased from Microbix Biosystems (Mississauga, ON, Canada). All the following viral antigens were inactivated and should not contain infectious material. Monolayers of cells were infected in a manner which ensured that the full host of viral antigens was present upon harvest. Cellular debris was removed. Human Cytomegalovirus HCMV, Grade 2 (Cat # EL-01-02), Parainfluenza virus Grade 2 (Cat #EL-09-02-001), Influenza virus Grade 2 (Cat # EL-13-02-001), Varicella Zoster virus, Grade 2 (Cat # EL-03-02), Mumps virus Grade 2 (Cat # EL-06-02), Measles virus Grade 2 (Cat # EL-04-02) and Rubella virus K1S Grade Antigen (Cat # EL-05-10). All bacterial antigens were inactivated and lyophilized native organisms. Microbix also provided one bacterial antigen, namely *Mycoplasma pneumoniae* (Cat # EL-11-02). All other bacterial antigens were purchased from Presque Isle Cultures & Labs (Erie, PA): *Lactobacillus bulgaris* (Cat # 546), *Escherichia coli* (Cat # 336), and *Streptococcus salivarius* (Cat # 518). Our test library additionally included *Candida albicans*, also from Presque Isle Cultures & Labs (Cat # 925). The CEF peptide pool consisting of 32 HLA Class 1-restricted peptides [1] widely used as a positive control for CD8 cell activation, was from CTL (CEFpp+, Cat. # CTL-CEF-002). The CEF peptide pool was added to the ELISPOT test system at a final concentration of 0.25 µg/mL. For all other antigens, optimal concentrations have been established (Figure S1). The CPI pool as a mix of three viral antigens, namely CMV, influenza and parainfluenza, is from CTL (Cat. # CTL-CPI-001) and was used at 6.25 µg/mL. Peptide pools covering HCMV different protein antigens of HCMV were from the PepMix™ Collection of JPT (Berlin, Germany): IE-1, IE-2, UL28, UL32, UL36, UL55, UL82, UL94, UL103, US3. Each of these peptide pools covered the amino acid sequence of the respective antigen by overlapping peptides of 15 amino acids in length, progressing in steps of five amino acids. All peptide pools were tested at 0.25 µg/mL.

### 2.3. Human Cytokine ELISPOT Assays

The human interferon-γ ELISPOT assay was performed as previously described using the ImmunoSpot^®^ Test Kit (CTL, Cat # hIFNG-1M) following the manufacturer’s protocol, and as described in [12]. In brief, the PVDF membrane was coated with a capture antibody overnight. Antigens were plated first in CTL Test Medium^TM^ (CTL, Cat #CTLT 005) in a volume of 100 μL per well at 2× the final concentrations. The plates containing the antigens were stored at 37 °C in a humidified CO_2_ incubator until the PBMC were ready for plating [13]. The thawed PBMC were suspended in CTL Test Medium^TM^ and added at 2.5 × 10^5^/cells per well in 100 μL—unless specified differently—using wide-bore pipette tips. Plates were gently tapped on each side to ensure an even distribution of the cells while settling onto the membrane, and then they were incubated at 37 °C in a humidified incubator at 9% CO_2_ for 24 h. After decanting the cells, the detection antibody was added and the membrane-bound cytokine was visualized by an enzymatic reaction. ELISPOT assays detecting IL-2, IL-4, IL-5, and IL-17 were performed in the same way using the respective kits for IL-2 (cat.# CTL-HIL2-1/5), IL-4 (cat.# HIL4-1/5), IL-5 (cat.# HIL5-1/5) and IL-17 (cat.# CTL- HIL17-1/5). Due to the delayed cytokine secretion kinetics of IL-4, IL-5 and IL-17-producing CD4 cells [14], the antigen stimulation culture for the IL-4 assays was 48 h, and for IL-5 and IL-17 assays, 72 h. The plates were air-dried in a laminar flow hood prior to analysis. The ELISPOT plates were scanned and analyzed using an ImmunoSpot^®^ S6 Ultimate Reader from CTL. The numbers of spots (Spot Forming Units, SFU) were automatically calculated by the ImmunoSpot^®^ Software (CTL) for each antigen stimulation condition vs. the medium (negative) control using SmartCount™ and Autogate™ functions [15]. In all experiments and for all donors, the negative control wells had less than 10 SFU per well. Spot counts reported for the respective antigen-stimulated test conditions are means of triplicate wells, without the medium control subtracted. As ELISPOT counts follow Gaussian (normal) distribution among replicate wells [16] permitting the utilization of parametric statistics, we performed Student’s t-tests for identifying positive responses. A *p* value < 0.05 was considered as the cut-off for positivity.

## 3. Results

### 3.1. Identifying Recall Antigens that Elicit IFN-γ Production in the Majority of Healthy Human Donors

**Figure 1:**
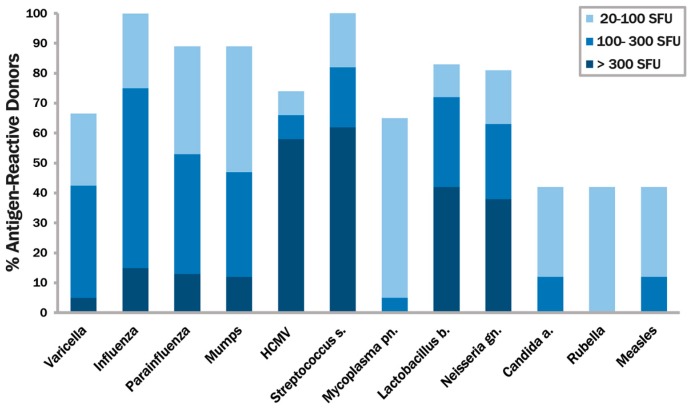
Initial screening of 16 donors with 12 ubiquitous antigenic systems. The antigens specified on the x-axis were tested on peripheral blood mononuclear cells (PBMC) of 16 donors in an interferon (IFN)-γ ELISPOT assay. The percentage of PBMC donors responding to each antigen is shown while also grading the magnitude of the response as specified.

We sought to identify environmental antigens to which most healthy humans are likely to have been exposed to, and to have developed immunity to, by the time they reach adulthood. Among viruses we selected varicella, influenza, parainfluenza, mumps, cytomegalovirus, rubella and measles. Among bacteria, *Streptococcus salivarius*, *Lactobacillus bifidus*, and *Mycoplasma pneumoniae* were selected. Our test library also included *Candida albicans*. Inactivated organisms, or their protein extracts were tested, as specified in Materials and Methods. For the initial screening, PBMC of 16 healthy donors were randomly selected. The numbers of IFN-γ producing cells were measured in a standard ELISPOT assay after challenging the PBMC of each donor with each of the above antigens. Figure 1 shows how many percent of these 16 donors’ PBMC responded with IFN-γ production to each of the antigens, with graded response levels specified. Varicella, Influenza, Parainfluenza, Mumps, HCMV, *Streptococcus*, and *Lactobacillus* all induced high to mid-level IFN-γ-producing cells in at least 50% of the test subjects. These are highlighted in the figure, and were selected for the subsequent studies.

**Figure 2:**
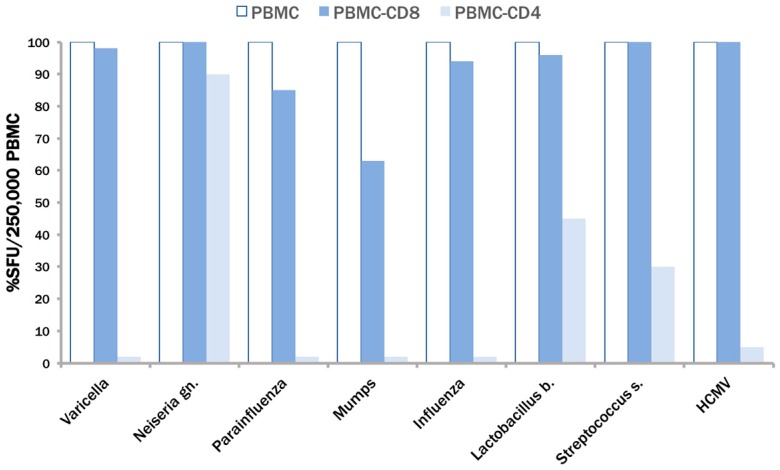
Establishing the CD4/CD8 lineage of responding PBMC. Unseparated PBMC (open bars), PBMC of the same subject that have been depleted of CD8 cells (blue bars), or of CD4 cells (light blue bars) were tested at the same cell number (250,000) per well. The numbers of IFN-γ producing cells were measured by ELISPOT after the addition of the antigens specified on the x-axis. Percent response in the depleted PBMC fractions is shown relative to the unseparated PBMC. Note, for varicella, parainfluenza, mumps and influenza the light blue bars are barely/non visible due to the close to complete abrogation of Spot Forming Units (SFU) formation in CD4 cell-depleted PBMC.

As stated in the Introduction, the uptake of extracellular proteins channels antigens towards the HLA-Class II antigen presentation pathway. Therefore, it seemed likely that the above antigens we used stimulated CD4 cells to produce IFN-γ. Working with complex antigens, including entire inactivated virions, however, also entailed the possibility that cells of the innate immune system become activated in addition to CD4 cells. We therefore performed cell separation experiments to identify the type of cell within the PBMC that produces IFN-γ after stimulation with these antigens. Unseparated PBMC were tested, in addition to PBMC that were depleted of either CD4 cells, or CD8 cells. As shown in Figure 2, CD4 cell depletion completely abrogated the IFN-γ production induced by Varicella, Parainfluenza, Mumps, Influenza, and HCMV. For these antigens, depletion of CD8 cells had either no effect (varicella, influenza, HCMV), or a weak effect (parainfluenza and mumps). These antigen preparations, therefore, primarily (or close to exclusively) stimulated CD4 cells and seemed to be suitable for developing a positive control for CD4 cells. In contrast, the depletion of CD4 cells reduced, but did not abrogate IFN-γ production induced by protein extracts of *Lactobacillus* and *Streptococcus* whereas CD8 cell depletion had no effect. These bacterial antigens did not prove to be suitable as a CD4 positive control, because in addition to stimulating CD4 cells, they also elicited IFN-γ production in cells of the innate immune system. The *Neisseria*-triggered IFN-γ production resulted entirely from the activation of non-CD4, non-CD8 cells.

### 3.2. None of the Recall Antigens Tested Individually Suffices to Trigger CD4 Recall Responses in All Subjects

Based on the above results, we narrowed in on Influenza-, HCMV-, Parainfluenza-, Mumps- and Varicella viruses as candidate antigens for a CD4 T cell positive control. The optimal antigen dose was established by titrating each of these antigens in IFN-γ ELISPOT assays using PBMC of three donors that gave high, intermediate, and low spot counts when stimulated with the antigen. It was 25-, 30-, 3.5-, 50- and 12.5 μg/mL for Influenza, HCMV, Parainfluenza, Mumps, and Varicella, respectively (Figure S1). Using the respective optimal antigen doses, we sought to establish the ideal PBMC number per well by titrating these cells. As shown in Figure S2, between 200,000 and 500,000 PBMC per well, a close-to-perfect linear relationship was seen between cell numbers plated and the SFU counts per well, for all five antigens (R^2^ > 0.95). For the further PBMC screenings we therefore continued with 250,000 PBMC per well.

With these optimized test conditions, we set out to test 100 healthy human donors, asking how frequently the individual antigens induced CD4 recall response in a larger test population. The CEF peptide pool that is widely used as a positive control for CD8 cells was tested in parallel on these PBMC samples. This comparison was sought because a CD4 cell positive control would need to perform at least as well, ideally better, as the present gold standard positive control for CD8 cells. As shown in Figure S3 with graded response levels, none of the individual antigens, including the CEF peptide pool, induced a recall response in more than 80% of the donors. Recall responses induced by Influenza (77%), Parainfluenza (77%), and HCMV (73%) were the most prevalent, each in a similar range as was found for the CEF pool (69%). Negative responses are highlighted in the figure. A closer look at the data revealed that donors who did not respond to one of the CD4 antigens did respond to several other antigens. While any combination of two of the above three prevalent antigens did not suffice to reach 100% coverage, the cumulative recognition of all three seemed promising.

### 3.3. The CPI Pool Triggers CD4 Cell Recall Responses in 100% of Healthy Donors

**Figure 3:**
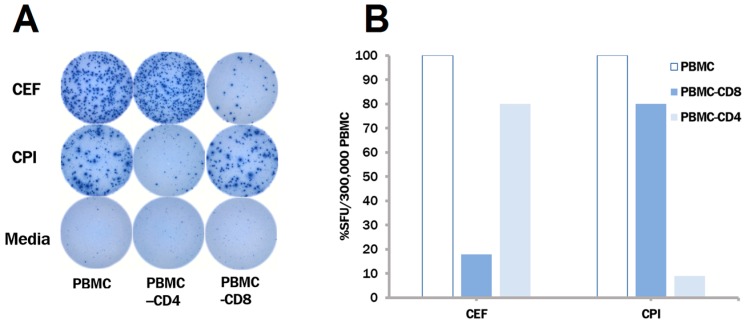
CPI (inactivated cytomegalo-, parainfluenza-, and influenza virions) recalls CD4 cells, CEF, (peptides of Cytomegalo-, Epstein-Barr and Flu-virus) recalls CD8 cells, respectively. Unseparated, as well as CD4- and CD8 cell-depleted PBMC of the same donor were tested for the CEF and CPI recall response, as specified. Representative well images are shown in (**A**). In (**B**) the percentage of response in the depleted PBMC fractions is shown relative to the unseparated PBMC.

Based on the above results it seemed that from combining inactivated cytomegalovirus, parainfluenza and influenza viruses into a single antigen pool that we named the CPI pool could elicit CD4 recall responses in 100% of healthy human donors, provided that the three antigens do not interfere with each other under the test conditions. To test this hypothesis, a CPI pool was created with the concentrations of each of the three antigens at the optimum, as established above. The CPI pool was tested—like its individual ingredients before—whether it still triggers primarily CD4 cells. As before, unseparated PBMC, and PBMC depleted of CD4 or CD8 cells were tested for their recall response to the CPI and CEF pools. As seen in Figure 3, the CPI pool elicited IFN-γ production in the unseparated PBMC and in the CD8 cell-depleted PBMC only, but not in CD4 cell-depleted PBMC. The reverse was seen for the CEF pool, establishing that the former activates CD4 cells, and the latter, CD8 cells.

**Figure 4:**
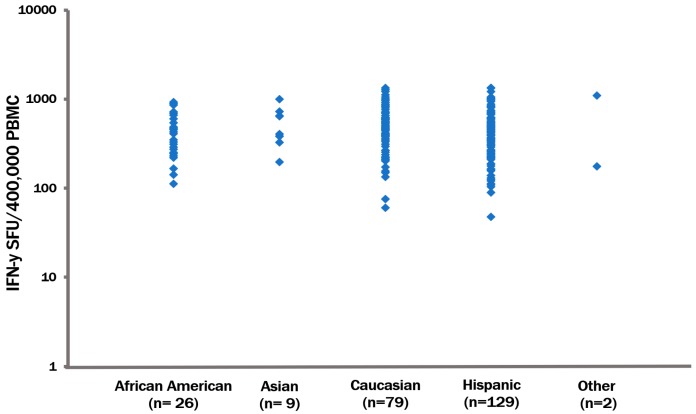
CPI recall responses in healthy donors. We tested 245 PBMC donors that have been grouped by race. The gender distribution in each group is specified in the text. The CPI-induced recall response of each donor is represented by a dot corresponding to the mean of three replicate wells. The average coefficient of variation calculated form these triplicate wells for each donor did not exceed 10%.

To test the hypothesis that the CPI pool is suited indeed as a universal CD4 cell control we obtained PBMC from 245 healthy adult donors and these were tested for their recall response to the CPI pool. The CEF peptide recall response was also tested, in parallel. These donors were randomly recruited by a blood bank in Southern California, thus the distribution of races and ethnicities is reflective of that area. Of the test subjects 79 were Caucasian (58 males, 21 females), nine Asian (eight males, one female), 26 were African American (19 males, seven females), 129 Hispanics (85 males, 44 females), one American Indian male, and one female. The results are summarized in Figure 4. All 245 donors generated a mid- to high level recall response to CPI, and even the weakest of which exceeded 60 SFU over a medium background of less than 10 SFU. The CPI response shown in Figure 3 is representative for such a week recall response. Most donors responded with over 100 SFU per 400,000 PBMC, and many exceeded 500 SFU, at which point the spots became confluent up to being too numerous to count (TNTC).

**Figure 5:**
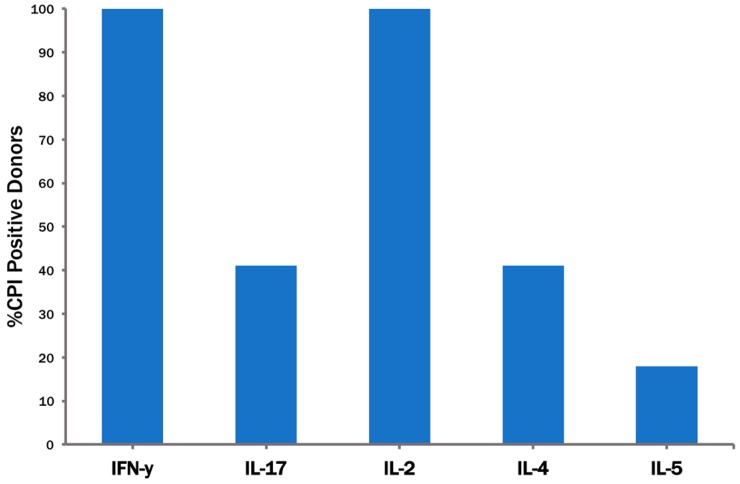
Th1, Th2 and Th17 lineage of CPI-reactive CD4 cells. Seventeen healthy donors were tested at 400,000 PBMC/well in IL-2, IL-4, IL-5, and IL-17 ELISPOT assays for the CPI-induced recall response. The percentage of PBMC donors displaying a positive cytokine recall response is shown.

Of note, the 100% CPI response rate was seen measuring IFN-γ only, suggesting that these donors have all developed Th1 immunity to the antigens contained in CPI. Th2 and Th17 cells [17] specific for CPI were seen only in a subset of donors (Figure 5): Seven of these 17 donors (41%) displayed a CPI-induced IL-4 and IL-17 recall response, and three out of 17 (17%) responded with IL-5. This outcome is not surprising, as we have obtained similar results in a systematic dissection of the HCMV-specific CD4 T cell response in healthy donors [12].

### 3.4. In Contrast to CEF, CPI Pool Provides Comprehensive Determinant Coverage for the Detection of T Cell Memory

Table 1 shows a side-by-side comparison of the CPI vs. CEF responsiveness of individual donors. While approximately half of the donors exhibited strong (>100 SFU per 400,000 PBMC) recall responses to both positive control pools, also in the above large-scale screening of 245 donors only 79% were CEF positive (of whom results for 40 random donors are shown in Table 1). Such negative CEF peptide responses are highlighted in yellow in Table 1. Several other donors generated a weak CEF recall response (between 10 and 30 SFU per 400,000 PBMC): these are highlighted in light yellow in Table 1. Strikingly, all of the CEF negative donors displayed strong recall responses to the CPI pool. The likely explanation of this finding is insufficient coverage of HLA-class I restricted determinants in the CEF pool, as discussed in the Introduction. Experimental evidence supporting this notion was sought. We argued that a CEF peptide pool negative response would imply the absence of CD8 cell memory to HCMV, provided the HCMV peptides contained in the CEF pool indeed provide sufficient determinant coverage for the HLA alleles present in the test population. This hypothesis was tested on six donors that all were CEF negative, but three of whom displayed a CD4 recall response to inactivated HCMV Gr2 antigen, while the other three did not respond to HCMV Gr2 antigen (Table 2). Negative responses are highlighted in the table. All three of the HCMV Gr2 antigen positive donors also responded to the majority of 11 peptide pools that covered 11 distinct open reading frames of HCMV. These three donors, therefore, beyond any doubt have developed strong T cell immunity to HCMV, yet were non-reactive to the CEF peptide pool. Therefore, the CEF peptide pool does not contain the HCMV peptides that would recall HCMV-reactive CD8 cells in these donors. The three donors in Table 1 who did not respond the HCMV Gr2 antigen also did not respond to the 11 HCMV peptide pools, suggesting that these donors, indeed, did not envelop T cell immunity to HCMV. Yet, while being non-responsive to CEF, these latter three donors also responded vigorously to CPI: as these latter three donors were apparently naïve to HCMV, their CPI recall response must have targeted either the parainfluenza, or the influenza, or both constituents of CPI. These results prove that the CEF peptide pool’s relatively frequent failure to stimulate a recall response results from its insufficient peptide coverage of the three viruses. In contrast, with the CPI pool all determinants for HLA-class II restricted antigen recognition are generated through natural antigen processing and presentation avoiding gaps in the peptide-determinant coverage for the detection of the antigen-specific CD4 cells.

## 4. Conclusions

We show here that 245 healthy adults of different races and ethnicities in Southern California generate a CD4 cell recall response to a combination of three viral antigens, cytomegalo-, parainfluenza- and influenza virus. In contrast, only 69% of these subjects responded to the CEF peptide pool. The presence or absence of a CD4 recall response to large protein antigens such as CPI is defined by antigen exposure itself, and not by the HLA-type. In contrast, the use of select peptides within the CEF pool for detecting CD8 recall responses is, in addition to antigen exposure, limited by the HLA-type of the test subject. While, based on antigen exposure, the CPI combination appears to be a close fit for adults living in Southern California, it will need to be learned whether the same antigen combination is suitable to provide 100% coverage for adult living elsewhere. As infections with Cytomegalovirus, Parainfluenza virus and Influenza virus first occur in childhood or early adulthood, one might expect that CPI will not be suited as a functional CD4 cell control early in life.

## Figures and Tables

**Table 1:** CEF peptide negative PBMC donors also respond to CPI. The CEF and CPI-induced recall response is shown for the specified 40 donors randomly selected from the total of 245 tested. Mean SFU counts for three replicate wells are shown. The medium background was less then 10 SFU for all donors (not shown). Negative recall response to CEF are highlighted in yellow, weak responses (that do not qualify for a positive control) are in light orange.

Donor-ID	CPI	CEF	Donor-ID	CPI	CEF	Donor-ID	CPI	CEF	Donor-ID	CPI	CEF
**LP_235**	810	112	**LP_260**	462	362	**LP_285**	268	83	**LP_313**	333	29
**LP_236**	1013	2	**LP_261**	725	600	**LP_286**	210	375	**LP_314**	600	161
**LP_237**	746	84	**LP_262**	155	25	**LP_288**	167	51	**LP_315**	163	22
**LP_238**	491	1	**LP_263**	1220	323	**LP_290**	223	7	**LP_316**	393	116
**LP_239**	843	4	**LP_264**	999	557	**LP_292**	240	352	**LP_317**	188	4
**LP_240**	619	509	**LP_265**	585	155	**LP_293**	224	138	**LP_318**	248	18
**LP_241**	563	287	**LP_266**	369	91	**LP_294**	268	58	**LP_319**	273	32
**LP_242**	957	41	**LP_267**	550	123	**LP_295**	500	101	**LP_320**	89	2
**LP_243**	125	55	**LP_268**	433	591	**LP_297**	260	11	**LP_321**	600	99
**LP_244**	536	358	**LP_269**	439	346	**LP_298**	215	208	**LP_322**	231	0

**Table 2:** Recall response to the CEF pool in comparison to the Human Cytomegalovirus (HCMV Gr.2 antigen and 11 different HCMV peptide pools. Six PBMC donors were selected that were CEF recall negative but either responded to HCMV Gr antigen (*n* = 3) or did not respond to HCMV Gr 2 antigen. All donors were tested additionally for reactivity to 11 peptide pools that each cover different HCMV antigens. Mean SFU counts for three replicate wells are shown. Negative recall response to the specified antigens are highlighted in yellow, borderline responses in light orange.

Donor-ID	Media	CPI	CEF	Gr.2	HCMV Peptide Pools										
					pp65	IE-1	IE-2	UL28	UL32	UL36	UL55	UL82	UL94	UL103	US3
**LP_099**	2	829	5	661	565	46	29	91	928	77	42	53	0	38	587
**LP_193**	0	720	3	250	437	278	98	13	152	139	651	2	290	2	269
**LP_194**	1	262	3	27	136	395	10	208	40	50	19	2	8	10	37
**LP_141**	3	322	2	0	0	5	0	0	0	0	2	2	0	0	0
**LP_151**	1	221	3	0	0	0	0	0	0	2	0	0	0	0	0
**LP_218**	0	266	0	0	2	0	0	2	2	0	0	0	2	0	0

## Supplementary Materials

The following are available online at www.mdpi.com/2073-4409/6/4/47/s1, Figure S1: Establishing optimal antigen concentrations for candidate antigens; Figure S2: Establishing the optimal PBMC number; Table S1: Test results for 100 donors screened for reactivity to the individual antigens.
