# Supplementary material for: A Positive Control for Detection of Functional CD4 T Cells in PBMC: The CPI Pool
Source: Cells. 2017 Dec 7;6(4):47. doi: 10.3390/cells6040047 (PMC5753071; doi:10.3390/cells6040047)
Supplement: Supplementary file 1 [file cells-06-00047-s001.pdf]

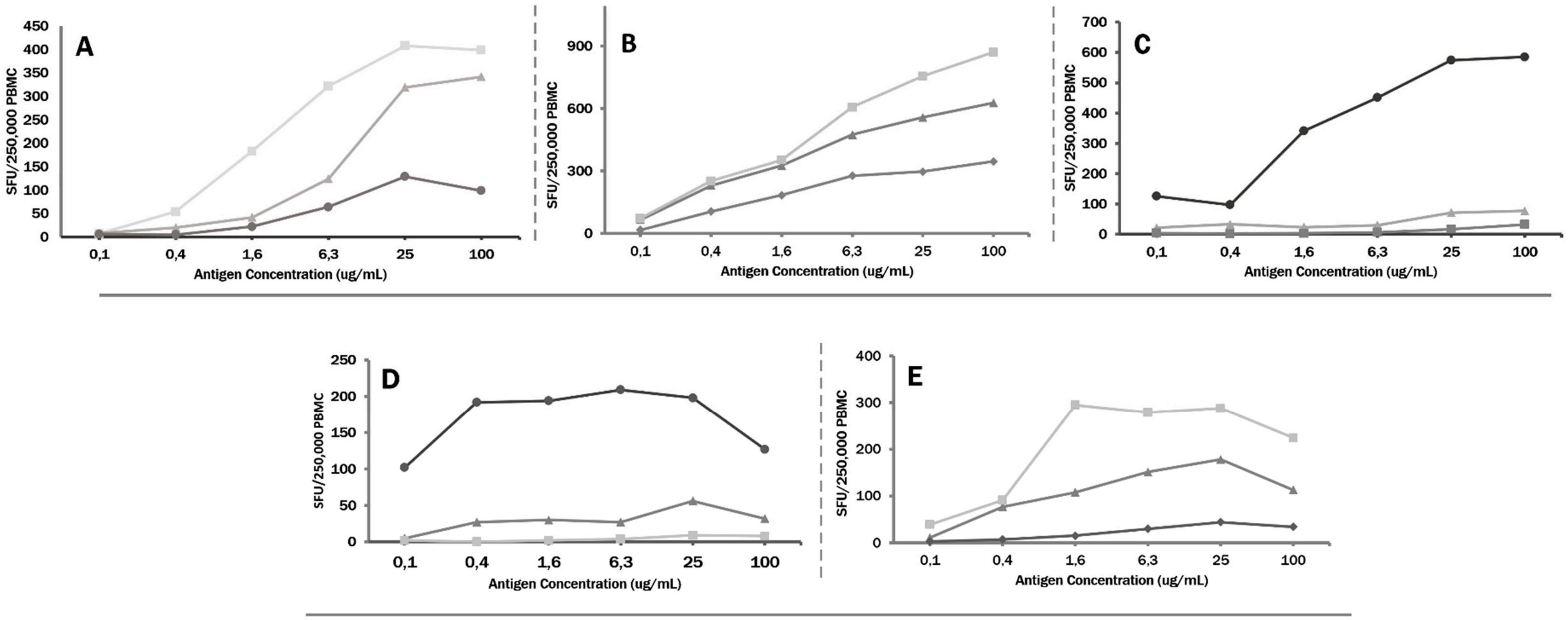

Figure S1: Establishing optimal antigen concentrations for candidate antigens. Antigen titrations are shown for influenza (Panel A), HCMV (Panel B) mumps (Panel C), varicella (Panel D), and parainfluenza (E). For each antigen PBMC of three donors were tested, distinguished by the symbols. The numbers of IFN-γ SFU elicited (Y axis) are plotted against the antigen concentrations (X axis). The concentration selected as optimal are: 25 μg/ml for influenza, 30 μg/ml for HCMV, 50 μg/ml for mumps, 12.5 μg/ml for varicella, and 3.3 μg/ml for parainfluenza.

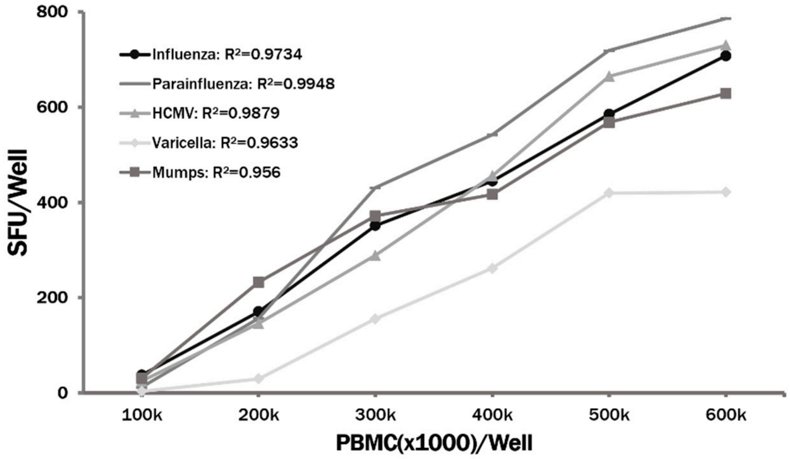

Figure S2. Establishing the optimal PBMC number. PBMC were plated in serial dilution in the number of cells specified on the X axis. The specified antigens were added at the optimal concentration established in Suppl. Fig 1. and the resulting number of IFN-γ SFU per well were measured (Y axis). The data show a close to linear relationship between cell numbers and spot counts from 200,000 to 500,000 PBMC per well: the R^2 values for linearity are specified.

Donor	Antigens					
	I	P	C	M	V	CEF
1	39	44	90	6	7	49
2	207	355	55	244	135	45
3	112	143	447	134	56	248
4	175	260	100	30	37	123
5	85	115	5	19	3	3
6	355	504	94	276	88	331
7	24	31	2	7	6	1
8	382	453	600	344	272	234
9	5	4	170	48	4	1
10	600	600	600	130	106	47
11	99	170	15	39	11	1
12	176	177	214	69	13	146
13	250	600	600	167	171	198
14	100	154	86	127	64	125
15	206	600	600	201	85	174
16	152	173	170	166	176	227
17	154	270	510	1	4	114
18	350	600	272	14	178	41
19	16	48	161	2	76	159
20	12	30	9	4	11	3
21	46	172	63	3	34	26
22	1	16	46	1	5	64
23	300	311	398	4	58	173
24	36	30	351	1	5	106
25	38	279	24	15	12	7
26	295	467	600	28	39	600
27	99	102	379	5	21	78
28	19	8	398	1	2	208
29	45	97	15	2	16	396
30	376	522	72	13	51	6
31	22	110	92	17	19	116
32	253	182	22	10	21	278
33	119	157	17	24	55	164
34	354	567	130	179	111	48
35	219	111	600	22	65	162
36	25	72	15	75	18	4
37	115	148	2	8	33	38
38	332	376	109	215	200	54
39	167	372	600	147	18	600
40	127	221	216	42	67	20
41	122	178	174	10	9	92
42	98	306	11	10	1	92
43	117	169	116	16	32	100
44	66	180	163	3	1	177
45	94	257	165	42	103	142
46	94	272	152	150	57	3
47	104	122	3	7	15	12
48	16	55	76	6	4	189
49	69	18	29	15	26	330
50	54	43	274	29	8	73
51	71	44	337	7	9	83

Donor	Antigens					
	I	P	C	M	V	CEF
51	71	44	337	7	9	83
52	83	37	3	5	13	71
53	162	246	177	69	22	51
54	309	144	122	17	24	266
55	143	369	475	115	89	68
56	14	5	73	5	2	262
57	53	35	50	29	17	190
58	31	31	44	27	8	3
59	445	402	408	516	478	291
60	266	31	152	41	35	118
61	8	12	24	12	23	10
62	23	8	59	8	4	101
63	113	34	3	18	4	40
64	85	96	145	14	22	268
65	29	44	4	44	17	187
66	92	145	33	120	36	48
67	22	22	525	156	25	4
68	45	167	243	172	8	104
69	223	117	84	113	35	570
70	22	4	0	14	0	4
71	14	22	1	23	1	24
72	77	9	0	38	2	2
73	25	69	185	74	29	2
74	14	12	24	42	10	2
75	395	561	277	463	53	235
76	93	94	174	277	71	309
77	58	24	90	40	24	8
78	52	39	38	87	24	128
79	65	11	2	39	4	4
80	307	138	331	345	51	303
81	305	66	13	249	10	85
82	177	65	2	91	9	283
83	5	13	86	59	5	13
84	169	50	17	361	28	85
85	179	106	447	650	51	52
86	224	47	6	127	20	261
87	74	27	1	50	7	4
88	9	40	3	65	3	105
89	452	406	9	222	27	20
90	33	31	276	214	22	12
91	70	140	8	600	8	8
92	74	427	762	152	29	5
93	325	452	17	600	25	85
94	114	34	136	135	19	336
95	166	124	473	174	15	401
96	42	8	111	72	5	600
97	81	298	67	217	97	17
98	162	71	223	185	14	0
99	38	50	12	150	15	12
100	11	27	19	53	10	0
%	77	77	73	65	52	69

Table S1. Test results for 100 donors screened for reactivity to the individual antigens.

"I" stands for influenza, "P" for parainfluenza, "C" for human cytomegalovirus, "M" for mumps virus, and "V" for varicella virus. These antigens and the CEF peptide pool were tested in an IFN-γ ELISPOT assay on the specified donors, with 250,000 PBMC per well. The results are shown as SFU per well highlighting negative responses in yellow.
